# Severity of the COVID‐19 pandemic assessed with all‐cause mortality in the United States during 2020

**DOI:** 10.1111/irv.12923

**Published:** 2022-01-19

**Authors:** F. Scott Dahlgren, Lauren M. Rossen, Alicia M. Fry, Carrie Reed

**Affiliations:** ^1^ CDC COVID‐19 Response Team Centers for Disease Control and Prevention Atlanta Georgia USA; ^2^ National Center for Health Statistics Centers for Disease Control and Prevention Hyattsville Maryland USA

**Keywords:** COVID‐19, public health, United States

## Abstract

**Background:**

In the United States, infection with SARS‐CoV‐2 caused 380,000 reported deaths from March to December 2020.

**Methods:**

We adapted the Moving Epidemic Method to all‐cause mortality data from the United States to assess the severity of the COVID‐19 pandemic across age groups and all 50 states. By comparing all‐cause mortality during the pandemic with intensity thresholds derived from recent, historical all‐cause mortality, we categorized each week from March to December 2020 as either low severity, moderate severity, high severity, or very high severity.

**Results:**

Nationally for all ages combined, all‐cause mortality was in the very high severity category for 9 weeks. Among people 18 to 49 years of age, there were 29 weeks of consecutive very high severity mortality. Forty‐seven states, the District of Columbia, and New York City each experienced at least 1 week of very high severity mortality for all ages combined.

**Conclusions:**

These periods of very high severity of mortality during March through December 2020 are likely directly or indirectly attributable to the COVID‐19 pandemic. This method for standardized comparison of severity over time across different geographies and demographic groups provides valuable information to understand the impact of the COVID‐19 pandemic and to identify specific locations or subgroups for deeper investigations into differences in severity.

## 1 INTRODUCTION

In the United States, infection with SARS‐CoV‐2 caused 380,000 reported deaths from March to December 2020.^1^ Reported deaths likely underestimate the true number of COVID‐19 deaths for a variety of reasons.^2^ To better reflect the mortality burden of the COVID‐19 pandemic, various estimates of excess deaths have been made, where excess deaths are defined as the difference between the observed numbers of deaths in a specific time period and expected numbers of deaths in the same time period based on mortality during prior years. These analyses of all‐cause mortality data found large relative increases in mortality during the pandemic among all age groups,^3^ but particularly people 25 to 44 years of age.^4^

In the United States and other temperate countries, overall mortality rates are generally highest during the winter months, with peak weekly mortality rates usually coinciding temporally with influenza virus activity.^5^ Vega and colleagues developed the Moving Epidemic Method (MEM), a useful analytical tool to compare the severity of influenza seasons over time, across countries, and across age groups that may have different baseline levels of morbidity.^6^, ^7^ The method sets standardized intensity thresholds based on the highest values of a surveillance indicator observed within recent, prior seasons. In this way, even though particular age groups or geographic areas have different baseline levels of mortality, the interpretations of the thresholds would be similar.

We adapted the MEM to all‐cause mortality data from the United States to assess the severity of the COVID‐19 pandemic across age groups and states. Because case ascertainment, definitions, testing practices, care seeking behavior, and clinical behavior have all changed in response to the pandemic,^8^, ^9^, ^10^ many of the available data sources with historical data for estimating severity, such as syndromic surveillance,^6^ are complicated by such pandemic‐influenced changes. Additionally, as SARS‐COV‐2 is novel, historical cause‐specific data are not available for comparison as they would be for influenza. Recent reports have documented that COVID‐19 was the third leading cause of death in 2020, accounting for more than two thirds of the increase in all‐cause mortality.^4^, ^11^, ^12^, ^13^ Overall death recording is unlikely to be affected by factors that have changed during the pandemic (e.g., changes in testing, case ascertainment, or care seeking behavior); these data may provide valuable information about the severity of the ongoing COVID‐19 pandemic. The purpose of this study was to set intensity thresholds in all‐cause death data from past years with the MEM and use those thresholds to describe the severity of the COVID‐19 pandemic in the United States over time and by age and by geography.

## 2 METHODS

### 2.1 Data

We used weekly counts of all‐cause mortality from January 2013 to December 2020 tabulated by the National Center for Health Statistics from the National Vital Statistics System. Data were stratified by age group: ≤18 years of age, 18–49 years of age, 50–64 years of age, 65–74 years of age, 75–84, and ≥85 years of age; and on jurisdiction of residence: the 50 states, District of Columbia, and New York City. The data were tabulated on May 15, 2021. We used Morbidity and Mortality Weekly Report weeks (MMWR weeks) to tabulate all‐cause mortality. A MMWR week begins on Sunday and ends on Saturday. The first MMWR week of a year is the week with at least 4 days in that year.

### 2.2 Adjusting for secular trends

Because of population growth and aging, it is important to account for long‐term trends in the numbers of deaths occurring in the United States. Because our methods relied on comparing recent data to historical data, we needed to adjust for any secular trends in all‐cause mortality. First, we truncated the original time series by removing weeks after September 28, 2019 to exclude both the 2019–2020 pneumonia and influenza season as well as the COVID‐19 pandemic. We decomposed these historical data into three components using local regression (LOESS): (1) a seasonal pattern, (2) a secular trend, and (3) the residuals.^14^ When added together, the three components of the decomposition are exactly the original data. Taken one at a time, the seasonal pattern is exactly the same each year; the secular trend slowly moves the seasonal pattern up and down; and the residuals are the unexplained variation in weekly mortality. Because the secular trend from this decomposition was defined only on the historical data, we needed to extrapolate the secular trend to the end of 2020. So, we fit a line to the secular trend from the decomposition using ordinary least squares and extrapolated this line to the end of the original time series. Next, we shifted this fitted line vertically such that its minimum was zero on the domain of the original time series to prevent taking the logarithm of a negative value later in our analysis. Finally, we added this line to the original time series to adjust for the secular trend over the entire study period.

### 2.3 Severity assessment

We used the adjusted time series for the severity assessment. We adapted methods from Vega and colleagues to calculate intensity thresholds, a part of their MEM.^6^, ^7^ Because mortality in the United States usually is at its highest during the fall and winter respiratory virus season, we considered the historical data by season (October to September), rather than calendar year. We used the 2013–2014 through the 2018–2019 seasons and identified the 3 largest values of weekly all‐cause deaths from each season. Then, we assumed these values followed a log‐normal distribution. We calculated three intensity thresholds corresponding to the 50th, 95th, and 99.5th percentiles of this distribution: the median, 1.6 standard deviations above the median, and 2.6 standard deviations above the median. We denoted the intensity thresholds as IT500, IT950, and IT995. These three thresholds defined the four severity categories: “low severity,” “moderate severity,” “high severity,” and “very high severity.” So, the peak weekly mortality has a 50% a priori probability of being low severity, 45% of being moderate severity, 4.5% of being high severity, and 0.5% of being very high severity. We used the intensity thresholds to assign severity of each week from week 10 through week 53 (March 1, 2020, through January 2, 2021) to one of the four severity categories. Compared with the original MEM, we used fewer peak weeks from each season and higher quantiles to define our ITs, resulting in universally higher ITs than the original MEM while preserving the original, relative spacing of ITs.^6^, ^7^ These changes reflect the unusually high severity of the pandemic and allow for distinguishing relatively unusually high severity in the setting of the pandemic. We repeated this analysis for the data stratified on age group and stratified on state of residence. Because data stratified on both age group and place of residence are often small and therefore redacted, we did not apply our methods to age groups within a jurisdiction.

### 2.4 Computer software

We used “R: a language and environment for statistical computing” for all computations.^15^ We also used the R package “MMWRweek: convert dates to MMWR day, week and year” to manage data.^16^

## 3 RESULTS

**FIGURE 1:**
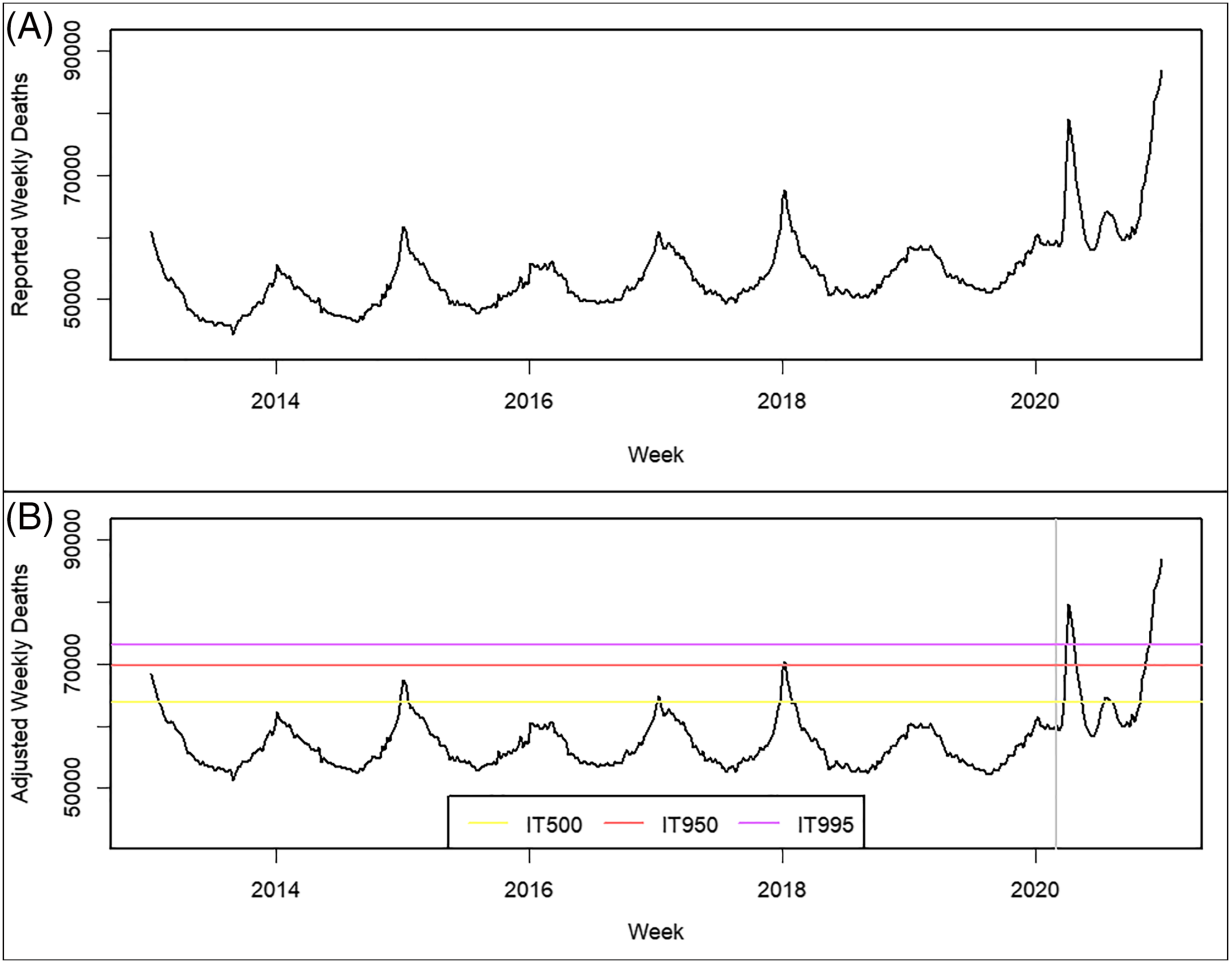
(A) A weekly time series of all‐cause mortality from January 6, 2013, through January 2, 2021. (B) The adjusted time series used to calculate the intensity thresholds (ITs), with the study period beginning at the vertical gray line on March 1, 2020. The difference between the two time series is the linear adjustment for secular trend

**FIGURE 2:**
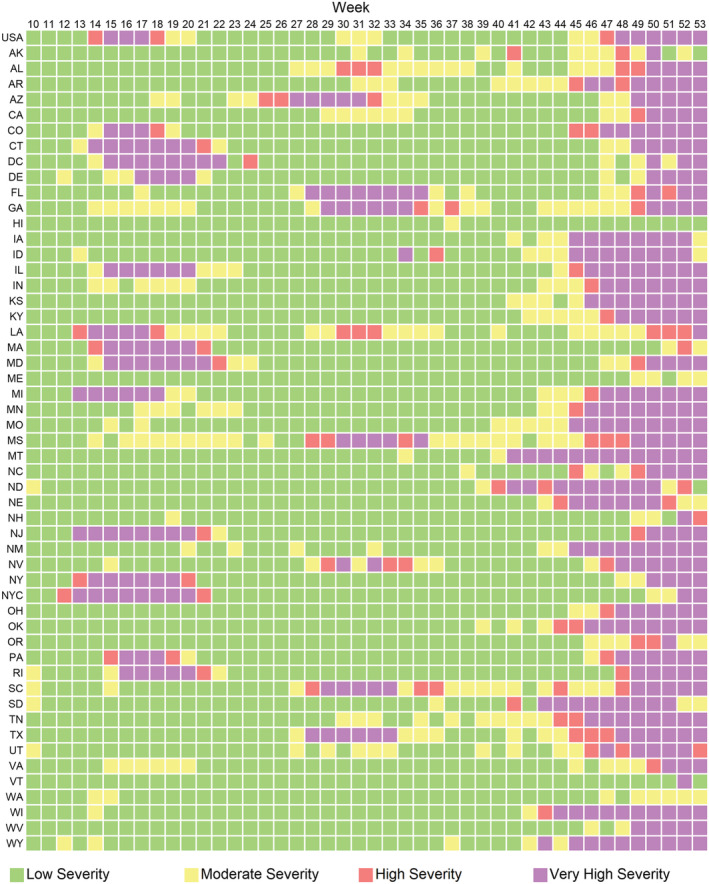
A plot of severity by state and by week from March 1, 2020, to January 2, 2021, assessed using weekly all‐cause mortality

From March 1, 2020, to January 2, 2021, nationally for all ages combined, all‐cause mortality was in the very high severity category for 9 weeks, from weeks 15 through 17 (April 5–April 25) and weeks 48 through 53 (November 22, 2020–January 2, 2021) (Figures 1 and 2). Forty‐seven states, the District of Columbia, and New York City each experienced at least one very high severity week for all ages combined (Figure 2). The average number of very high severity weeks per jurisdiction was 7.5 weeks, about 17% of the period from week 10 to week 53. There was variation in when states experienced very high severity and for how long. Overall, there were three distinct periods in which individual states experienced very high severity, in the spring during weeks 13–22 (March 22–May 30), in the summer from weeks 27–35 (June 28–August 29) (Figure 2), and in the fall/winter from weeks 41–53 (October 4–January 2). Some states experienced very high severity in multiple time periods, although most states did not experience very high severity until the fall/winter weeks. Hawaii, Maine, and Washington had no weeks of very high severity; Alaska, New Hampshire, Oregon, and Vermont had only 1 week.

**FIGURE 3:**
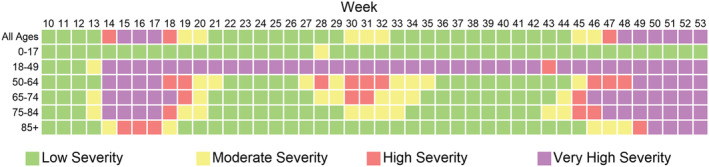
A plot of severity by age group and week from March 1, 2020, to January 2, 2021, assessed using weekly all‐cause mortality

Among children under 18 years old, all‐cause mortality was of moderate severity for week 28; all other weeks were low severity weeks for this age group; and none of the weeks were high or very high severity. All the adult age groups experienced some weeks of very high severity, though the number of weeks decreased with increasing age group, with 29 weeks of consecutive very high severity among adults 18 to 49 years old (Figure 3). Supplemental figures display time series for age groups (Figures S1–S6).

Supplemental figures illustrate the adjustment for secular trend (Figure S7), the values used to compute the ITs (Figure S8), and the statistical assumptions about the categories of severity (Figure S9).

## 4 CONCLUSIONS

All‐cause mortality during the COVID‐19 pandemic was unusually high compared with recent, historical all‐cause mortality. We attribute the very high severity of mortality during the study period to the COVID‐19 pandemic, though not all of the increase in all‐cause mortality may be directly attributable to infection with SARS‐CoV‐2; other causes may have been indirectly affected by the pandemic, including addiction‐related deaths and motor‐vehicle fatalities.^17^, ^18^, ^19^ Cause‐specific mortality data are not yet available by age and state, but in the future the MEM could be applied to specific categories of causes of death (e.g., respiratory, circulatory, injury, etc.) to observe which causes were more or less affected by the COVID‐19 pandemic and among which age groups and states.

This method is especially useful for comparing across geographies or age groups that have very different baseline levels of mortality. While weekly all‐cause mortality was highest among adults at least 65 years of age (Figure S1–S4), when comparing age groups to their own historical peak mortality rates, we noted a lengthy period of sustained very high mortality among younger adults, 18–49 years of age, with 29 consecutive weeks of all‐cause mortality in the very high severity level. As cause‐specific mortality rates become available, further investigation of cause‐specific mortality rates may help explain how this age group was impacted by the pandemic. During the COVID‐19 pandemic, or future public health emergencies, this method flags potential trends that would benefit from further exploration.

We also noted substantial geographic heterogeneity. The three periods of very high mortality across the states in the spring, summer, and fall/winter coincide generally with increases in reported COVID‐19 cases and deaths nationally.^20^ Some states experienced very high severity in multiple time periods, although most states did not experience very high severity levels until the fall/winter weeks. On average, jurisdictions experienced 7.5 very high severity weeks. Multiple jurisdictions experienced >10 very high severity weeks (Connecticut, District of Columbia, Florida, Illinois, Maryland, Michigan, Montana, New Jersey, and Texas); and a handful of states experienced ≤1 very high severity week (Alaska, Hawaii, Maine, New Hampshire, Oregon, Vermont, and Washington) (Figure 2). This may be another area where this method can be used to help target a deeper investigation of why some states had relatively more or less burden of the COVID‐19 pandemic.

This method does have some limitations. While all‐cause mortality among adults at least 50 years of age in the historical seasons had a strong seasonal pattern, mortality among children and adults 18 to 49 years of age did not. Because of the lack of seasonality, the highest mortality weeks may be spaced throughout the annual season instead of close together. However, the interpretation remains valid, in the sense that all‐cause mortality during the study period was unlikely to be highly elevated for several weeks, much less for 29 consecutive weeks. Our results rely on extrapolation of the secular trend from March 2020 to December 2020, and interpretation of our results should be more qualitative as the study period progresses. For future use of the MEM to analyze all‐cause mortality data, the inclusion of March to December 2020 in the historical data will increase both the mean and the variance of peak weekly mortality, resulting in ITs which are both higher and further spaced. Depending on the desired interpretation, adjustments to the MEM may be helpful when investigating particular counterfactuals and hypotheses.

It is perhaps not surprising, given the high number of reported COVID‐19 deaths, that unusually high mortality rates were observed in the United States during the COVID‐19 pandemic. By setting standardized statistical thresholds in the mortality data, however, this method allows for easy comparison across age groups, geography, and over time, even when the baseline mortality rates differ, to identify the time periods, subpopulations, and places that experienced the greatest standardized increases in deaths as a result of the pandemic. Furthermore, because this analysis relies on all‐cause mortality, a metric that is widely monitored and less likely affected by misclassification or reporting changes during the pandemic, similar comparisons could be made across countries if death registration data during 2020 and prior years were available. This method for standardized comparison of pandemic severity over time across different geographies and demographic groups provides valuable information to better understand the differential impact of the COVID‐19 pandemic across locations or subgroups. Results can inform future investigations into the factors that may have contributed to differences in the severity of the pandemic across populations in terms of relative increases in all‐cause mortality.

## AUTHOR CONTRIBUTIONS

**Fredrick Scott Dahlgren:** Writing‐original draft; formal analysis. **Lauren M. Rossen:** Writing‐review and editing. **Alicia M. Fry:** Conceptualization; writing‐review and editing. **Carrie Reed:** Conceptualization; writing‐review and editing.

## CONFLICT OF INTEREST

The authors have no conflict of interest to disclose.

## DISCLAIMER

The findings and conclusions in this report are those of the authors and do not necessarily represent the official position of the Centers for Disease Control and Prevention.

### PEER REVIEW

The peer review history for this article is available at https://publons.com/publon/10.1111/irv.12923.

## Supporting information


**Figure S1:** (**A**) A weekly time series of all‐cause mortality among people under 18 years of age from January 6, 2013 through January 2, 2021. (**B**) The time series adjusted for secular trend used to calculate the intensity thresholds (ITs), with the study period beginning at the vertical gray line on March 1, 2020.
**Figure S2:** (**A**) A weekly time series of all‐cause mortality among people 18 to 49 years of age from January 6, 2013 through January 2, 2021. (**B**) The time series adjusted for secular trend used to calculate the intensity thresholds (ITs), with the study period beginning at the vertical gray line on March 1, 2020.
**Figure S3:** (**A**) A weekly time series of all‐cause mortality among people 50 to 64 years of age from January 6, 2013 through January 2, 2021. (**B**) The time series adjusted for secular trend used to calculate the intensity thresholds (ITs), with the study period beginning at the vertical gray line on March 1, 2020.
**Figure S4:** (**A**) A weekly time series of all‐cause mortality among people 65 to 74 years of age from January 6, 2013 through January 2, 2021. (**B**) The time series adjusted for secular trend used to calculate the intensity thresholds (ITs), with the study period beginning at the vertical gray line on March 1, 2020.
**Figure S5:** (**A**) A weekly time series of all‐cause mortality among people 75 to 84 years of age from January 6, 2013 through January 2, 2021. (**B**) The time series adjusted for secular trend used to calculate the intensity thresholds (ITs), with the study period beginning at the vertical gray line on March 1, 2020.
**Figure S6:** (**A**) A weekly time series of all‐cause mortality among people at least 85 years of age from January 6, 2013 through January 2, 2021. (**B**) The time series adjusted for secular trend used to calculate the intensity thresholds (ITs), with the study period beginning at the vertical gray line on March 1, 2020.
**Figure S7:** The adjustment for secular trend in weekly all‐cause mortality among adults. The black line is a result from the decomposition of the historical data into: (1) a seasonal pattern, (2) a secular trend, and (3) the residuals.^13^ If we add the black line to the historical data, the resulting time series has no secular trend. The red line is the least squares fit of the black line, extrapolated past the historical data. Also, the red line is the difference between the time series in panel A and B of Figure 1.
**Figure S8:** The 3 largest values of each season from the historical time series of weekly all‐cause mortality among adults. These values directly determine the intensity thresholds (ITs) for categorizing severity.
**Figure S9:** The probability of the peak value of adjusted mortality from March 1, 2020 to January 2, 2021, using the moving epidemic method and the historical data on all‐cause mortality.

## Data Availability

The data are not publicly available due to privacy or ethical restrictions.
