# Supplementary material for: Severity of the COVID‐19 pandemic assessed with all‐cause mortality in the United States during 2020
Source: Influenza Other Respir Viruses. 2022 Jan 19;16(3):411–6. doi: 10.1111/irv.12923 (PMC8983917; doi:10.1111/irv.12923)
Supplement: Supplementary file 1 — Figure S1: (A) A weekly time series of all‐cause mortality among people under 18 years of age from January 6, 2013 through January 2, 2021. (B) The time series adjusted for secular trend used to calculate the intensity thresholds (ITs), with the study period beginning at the vertical gray line on March 1, 2020. Figure S2: (A) A weekly time series of all‐cause mortality among people 18 to 49 years of age from January 6, 2013 through January 2, 2021. (B) The time series adjusted for secular trend used to calculate the intensity thresholds (ITs), with the study period beginning at the vertical gray line on March 1, 2020. Figure S3: (A) A weekly time series of all‐cause mortality among people 50 to 64 years of age from January 6, 2013 through January 2, 2021. (B) The time series adjusted for secular trend used to calculate the intensity thresholds (ITs), with the study period beginning at the vertical gray line on March 1, 2020. Figure S4: (A) A weekly time series of all‐cause mortality among people 65 to 74 years of age from January 6, 2013 through January 2, 2021. (B) The time series adjusted for secular trend used to calculate the intensity thresholds (ITs), with the study period beginning at the vertical gray line on March 1, 2020. Figure S5: (A) A weekly time series of all‐cause mortality among people 75 to 84 years of age from January 6, 2013 through January 2, 2021. (B) The time series adjusted for secular trend used to calculate the intensity thresholds (ITs), with the study period beginning at the vertical gray line on March 1, 2020. Figure S6: (A) A weekly time series of all‐cause mortality among people at least 85 years of age from January 6, 2013 through January 2, 2021. (B) The time series adjusted for secular trend used to calculate the intensity thresholds (ITs), with the study period beginning at the vertical gray line on March 1, 2020. Figure S7: The adjustment for secular trend in weekly all‐cause mortality among adults. The black line is a result from [file IRV-16-411-s001.docx]

# SUPPLEMENTAL FIGURES


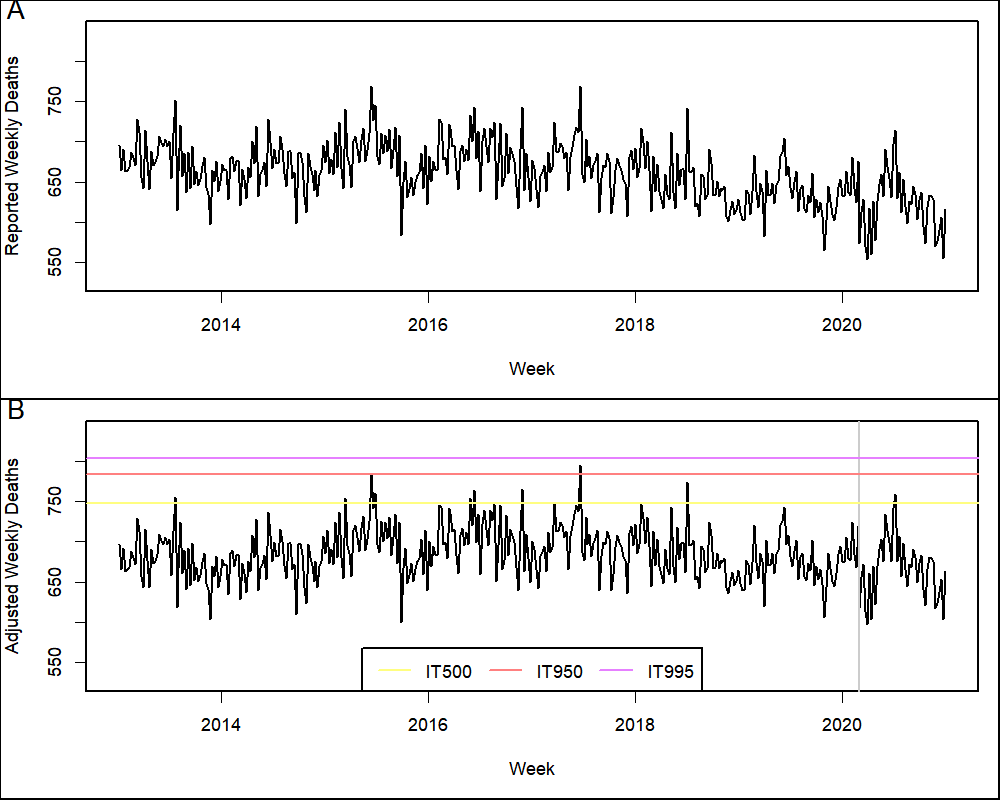


Figure S1: (**A**) A weekly time series of all-cause mortality among people under 18 years of age from January 6, 2013 through January 2, 2021. (**B**) The time series adjusted for secular trend used to calculate the intensity thresholds (ITs), with the study period beginning at the vertical gray line on March 1, 2020.


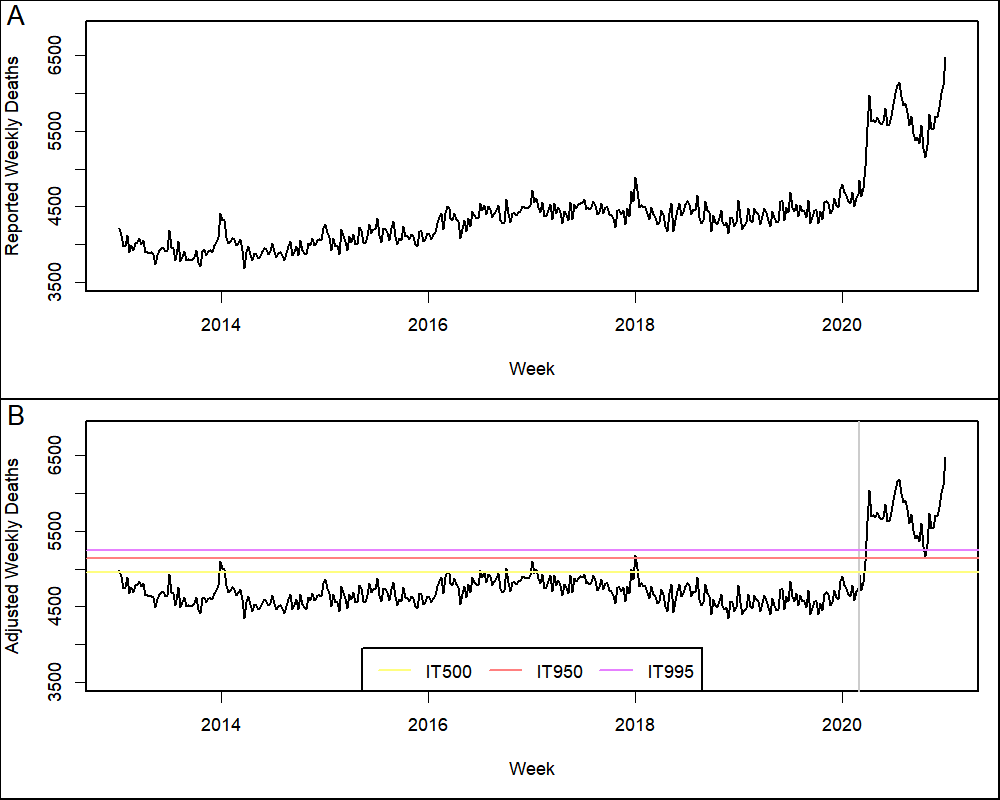


Figure S2: (**A**) A weekly time series of all-cause mortality among people 18 to 49 years of age from January 6, 2013 through January 2, 2021. (**B**) The time series adjusted for secular trend used to calculate the intensity thresholds (ITs), with the study period beginning at the vertical gray line on March 1, 2020.


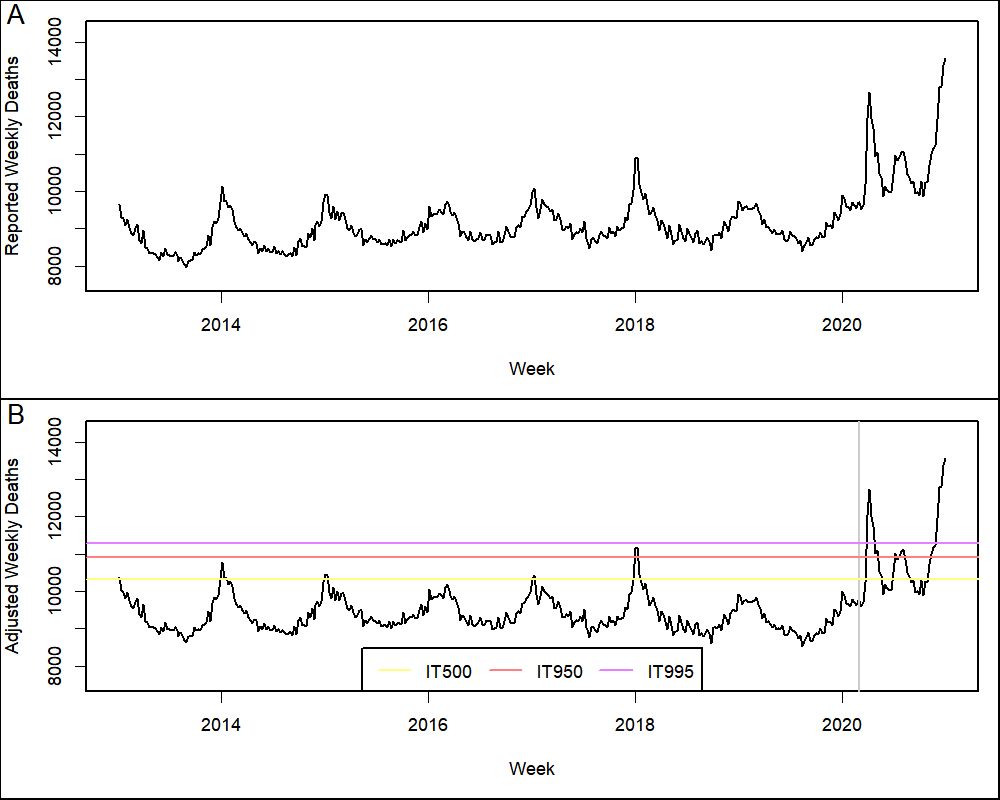


Figure S3: (**A**) A weekly time series of all-cause mortality among people 50 to 64 years of age from January 6, 2013 through January 2, 2021. (**B**) The time series adjusted for secular trend used to calculate the intensity thresholds (ITs), with the study period beginning at the vertical gray line on March 1, 2020.


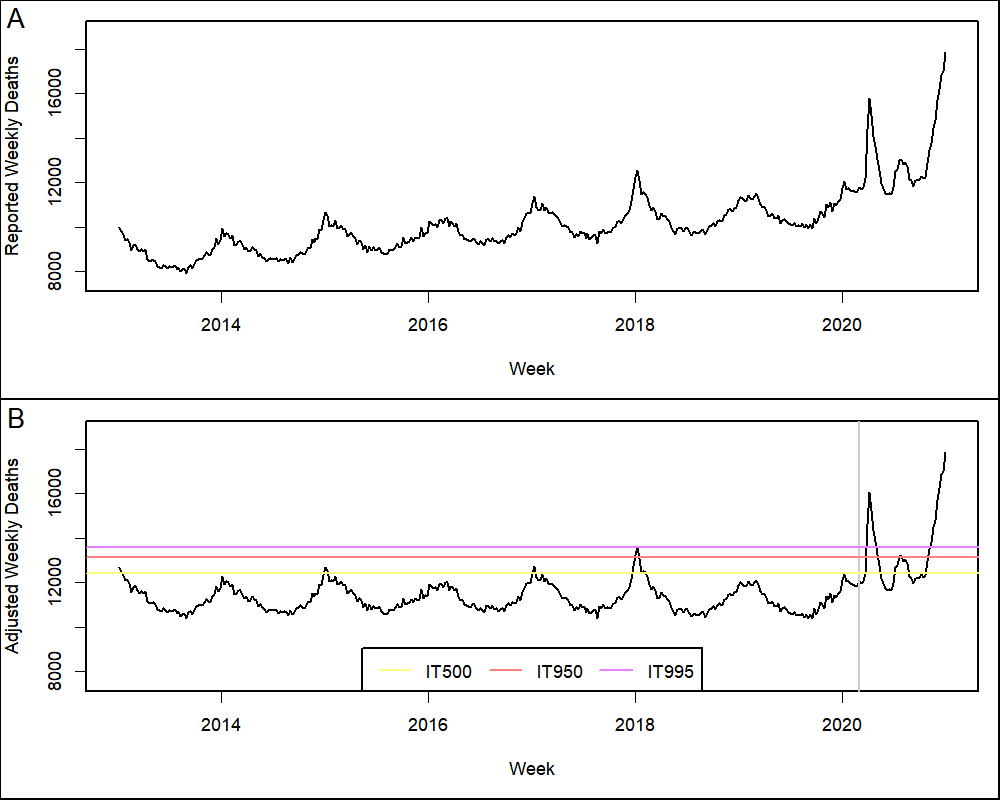


Figure S4: (**A**) A weekly time series of all-cause mortality among people 65 to 74 years of age from January 6, 2013 through January 2, 2021. (**B**) The time series adjusted for secular trend used to calculate the intensity thresholds (ITs), with the study period beginning at the vertical gray line on March 1, 2020.


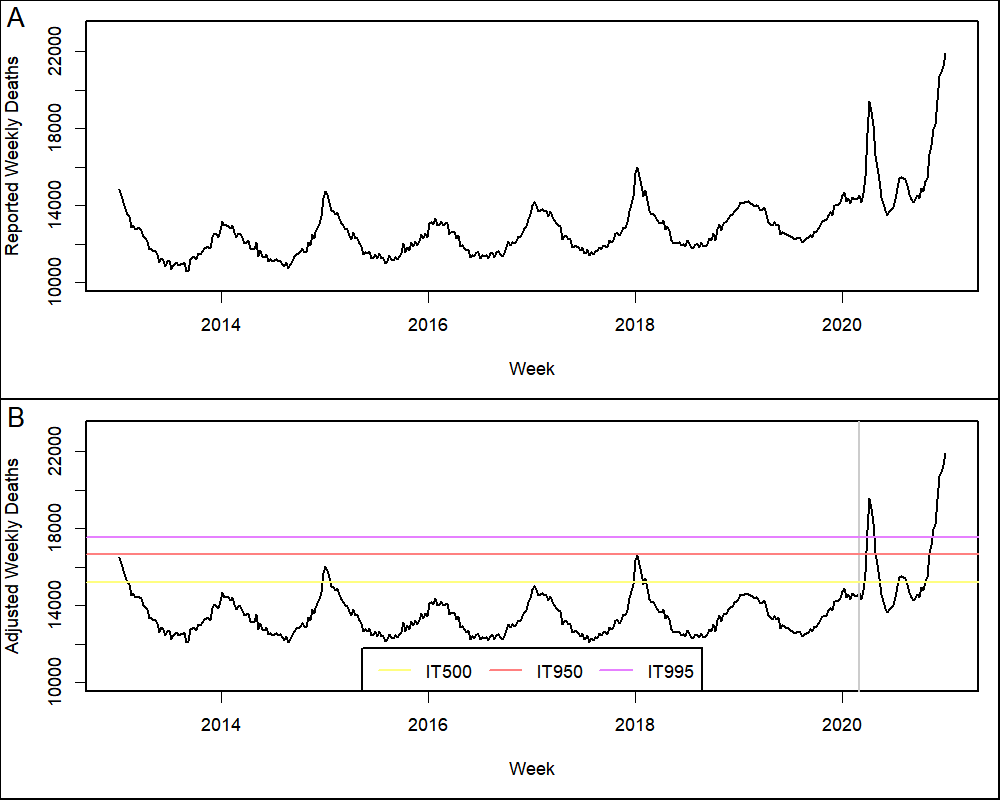


Figure S5: (**A**) A weekly time series of all-cause mortality among people 75 to 84 years of age from January 6, 2013 through January 2, 2021. (**B**) The time series adjusted for secular trend used to calculate the intensity thresholds (ITs), with the study period beginning at the vertical gray line on March 1, 2020.


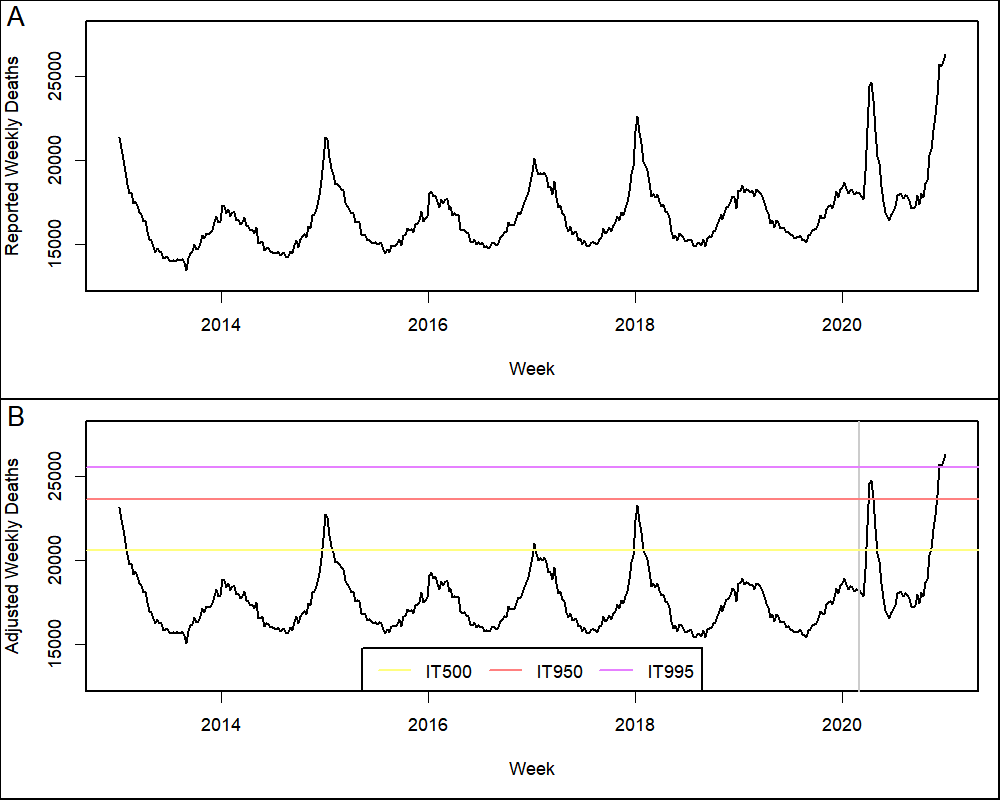


Figure S6: (**A**) A weekly time series of all-cause mortality among people at least 85 years of age from January 6, 2013 through January 2, 2021. (**B**) The time series adjusted for secular trend used to calculate the intensity thresholds (ITs), with the study period beginning at the vertical gray line on March 1, 2020.


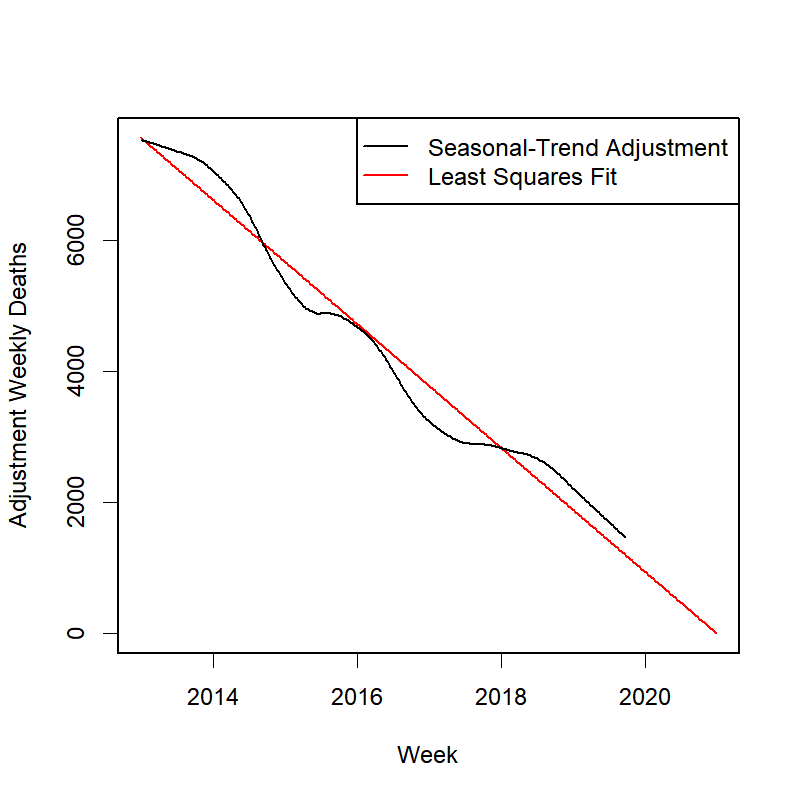


Figure S7: The adjustment for secular trend in weekly all-cause mortality among adults. The black line is a result from the decomposition of the historical data into: (1) a seasonal pattern, (2) a secular trend, and (3) the residuals.^13^ If we add the black line to the historical data, the resulting time series has no secular trend. The red line is the least squares fit of the black line, extrapolated past the historical data. Also, the red line is the difference between the time series in panel A and B of Figure 1.


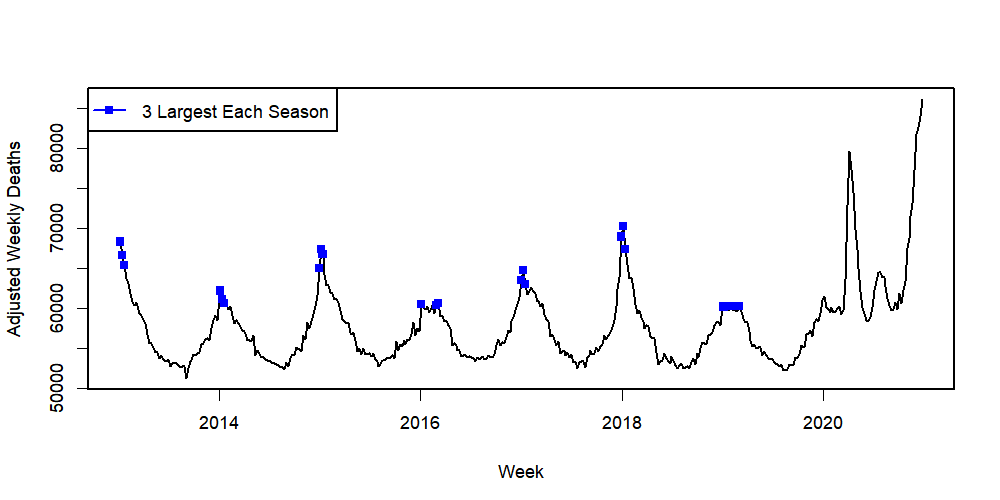


Figure S8: The 3 largest values of each season from the historical time series of weekly all-cause mortality among adults. These values directly determine the intensity thresholds (ITs) for categorizing severity.


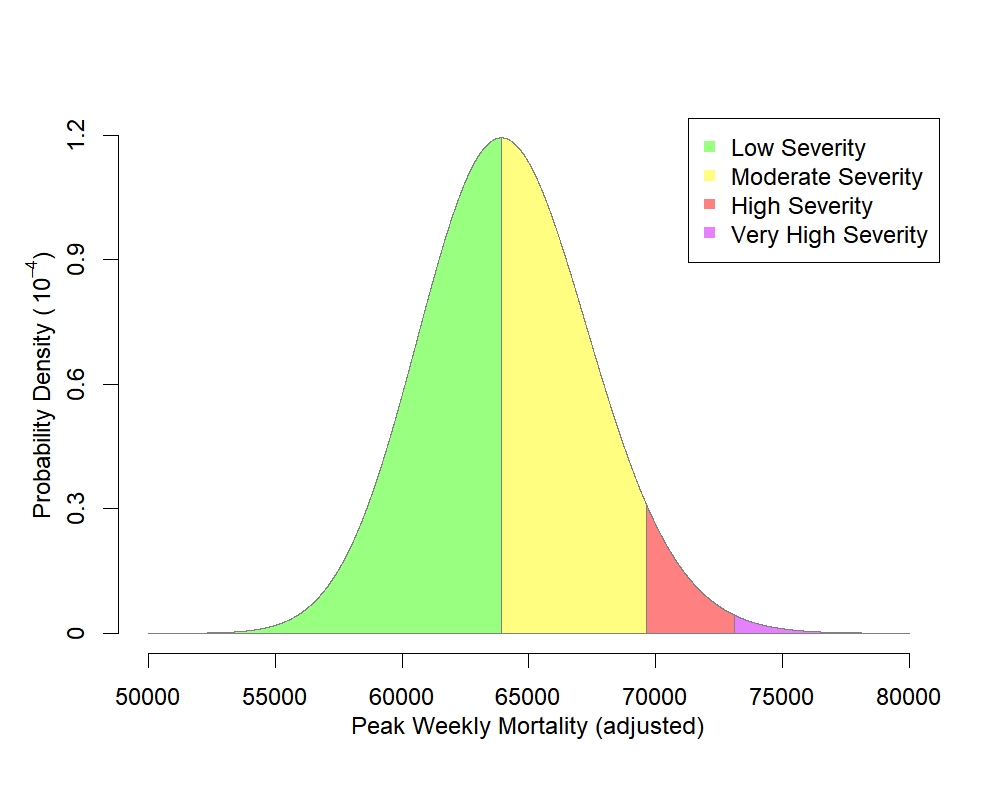


Figure S9: The probability of the peak value of adjusted mortality from March 1, 2020 to January 2, 2021, using the moving epidemic method and the historical data on all-cause mortality.
